# Macroscopic Pattern Formation of Alginate Gels in a Two-Dimensional System

**DOI:** 10.3390/gels9060444

**Published:** 2023-05-26

**Authors:** Ryota Haraguchi, Yushi Oishi, Takayuki Narita

**Affiliations:** Department of Chemistry and Applied Chemistry, Saga University, 1 Honjo, Saga 840-8502, Japan; 22804003@edu.cc.saga-u.ac.jp (R.H.); oishiy@cc.saga-u.ac.jp (Y.O.)

**Keywords:** alginate gel, pattern formation, phase separation, gelation, Liesegang phenomenon

## Abstract

Macroscopic spatial patterns were formed in calcium alginate gels when a drop of a calcium nitrate solution was placed on the center of a sodium alginate solution on a petri dish. These patterns have been classified into two groups. One is multi-concentric rings consisting of alternating cloudy and transparent areas observed around the center of petri dishes. The other is streaks extending to the edge of the petri dish, which are formed to surround the concentric bands between the concentric bands and the petri dish edge. We have attempted to understand the origins of the pattern formations using the properties of phase separation and gelation. The distance between two adjacent concentric rings was roughly proportional to the distance from where the calcium nitrate solution was dropped. The proportional factor *p* increased exponentially for the inverse of the absolute temperature of the preparation. The *p* also depended on the concentration of alginate. The pattern characteristics in the concentric pattern agreed with those in the Liesegang pattern. The paths of radial streaks were disturbed at high temperatures. The length of these streaks shortened with increasing alginate concentration. The characteristics of the streaks were similar to those of crack patterns resulting from inhomogeneous shrinkage during drying.

## 1. Introduction

Patterns appearing in hydrogel have attracted much interest among scientists. Their characteristics (morphology, size, and dynamics) are closer to those of living organisms [1,2,3,4,5]. Many physical hydrogels are formed from aqueous solutions of polysaccharides [6], which are commonly found in plant cell walls and have also been found in the extracellular matrix of animal cells. Typically, they form the skeletal structure of cellular tissues and act as compressive buffers against physical stress [7]. Most aqueous polysaccharide solutions form hydrogels by adding specific metal ions; for example, pectin and carrageenan form gels with calcium and potassium ions, respectively [8,9,10]. Some hydrogels formed by polysaccharides and their specific metal ions form macroscopic anisotropic structures. For example, Dobashi et al. demonstrated that a structured gel could be easily formed by dialyzing an aqueous sodium hydroxide solution (pre-gel solution) placed in a semipermeable membrane tube with aqueous calcium chloride (gelling agent) [11,12]. Core-shell structured gel beads are self-formed when a drop of Curdlan in aqueous sodium hydroxide gels by aqueous calcium chloride [13]. We have previously reported that striped cylindrical gels are obtained when potassium ions (gelling agent) are contacted with a glass tube filled with κ-carrageenan solution (pre-gel solution) from an end tube [14]. Such anisotropic structures result in the formation of polymer-rich and polymer-poor phases due to phase separation. Phase separations obtained in polysaccharide solution systems by adding specific metal ions often form gels simultaneously [15]; the gels appear cloudy or shrunken. These findings suggest that polysaccharide hydrogels formed by reaction–diffusion systems often have macro-anisotropic structures due to the competition of gelation. Limitations and conditions of metal ion diffusion during gelation dramatically change the macroscopic anisotropic structure to, for example, a core-shell shape or a stripe structure. Here, we studied macro-anisotropic structures formed when a drop of aqueous calcium nitrate (gelling agent) was placed in the center of petri dishes filled with alginate solution. Alginate is a polysaccharide consisting of the uronic acid β-d-mannuronate (M) and its C-5 epimer α-l-guluronate (G). The aqueous sodium alginate solution forms a thermally irreversible gel in the presence of divalent cations [9,16]. When two adjacent guluronate blocks are placed face to face, a crater forms where bivalent ions can be trapped; bivalent cations such as Ca^2+^ are the right size for this crater. When polymers link together through the Ca^2+^ coordination, it results in gelation through the formation of junction zones. The alginate–calcium complexes are insoluble in water, and calcium alginate gels tend to be unstable and can separate into gel and solution phases. If the polymer concentration is below a certain threshold, only clusters will form instead of continuous gels, resulting in a thick fluid [17]. Some alginate–calcium gels become opaque due to phase separation. The gel structure formed is synchronized with the phase separation and depends on several environmental conditions, such as temperature, pH, and component ratios [17,18,19]. The volume after gelation increases with increasing sodium alginate concentration and decreases with increasing gelation temperature. This effect may be due to the viscosity of sodium alginate, which increases with increasing concentration or decreasing gelation temperature [20]. Here, we report on a novel spatial pattern of calcium alginate gels self-assembled on petri dishes. We also demonstrate that the nature of the patterns depends on the gelation temperature and the alginate concentration of the pre-gel solution. The origins of the observed patterns are discussed based on the gelation coupling with phase separation and Liesegang phenomenon [21,22,23,24,25], in which concentric, regular rings of precipitates are formed when a certain reactant diffuses into a gel.

## 2. Results

**Figure 1:**
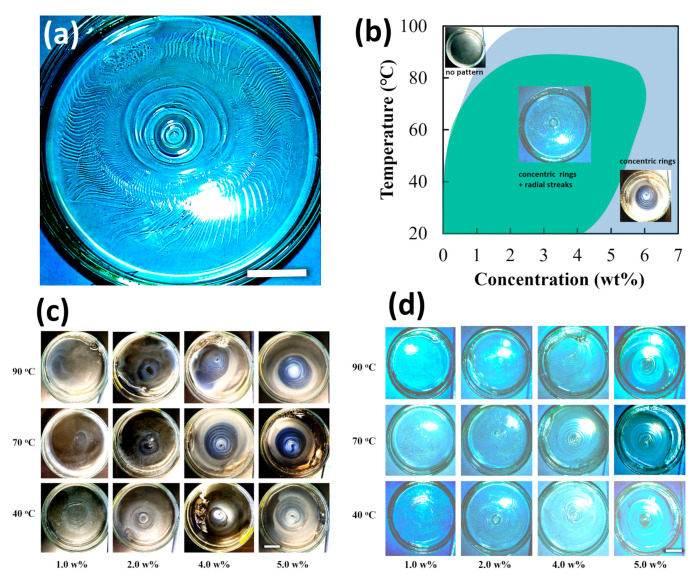
The morphology formed when sodium alginate solution in a petri dish was gelated by a calcium nitrate solution dropped in the middle: (**a**) a representative image of the alginate gel, prepared with alginate concentration 2.0 wt% at 40 °C; (**b**) morphological phase diagram of functions of the preparation temperature and alginate concentration; thumbnail images of alginate gels prepared at different temperatures and alginate concentrations on petri dishes photographed on (**c**) a black and (**d**) a white background. White bars represents 20 mm. Higher quality images of (**c**,**d**) are presented in Figure S1.

All samples formed gels within 24 h at different temperatures and alginate concentrations. Figure 1a illustrates a representative image of the gel formed on the petri dish. Almost all hydrogels had two types of macroscopic spatial patterns. These patterns held their forms for more than 1 month. One pattern was an alternating pattern of opaque and transparent concentric rings, which appeared in the central area of the petri dishes. The opaque areas were relatively thicker than the transparent ones. The adjacent rings had nearly the same width for both opaque and transparent. The ring widths increased along the radial direction from the center to the edge of the petri dish. The other spatial pattern, distinguished from the stripe pattern, was the radial streaks at the periphery of the concentric ring; they radiated outward from the center to the edge of the dish. These radial streaks became meandering at higher preparation temperatures, especially at low alginate concentrations. A high alginate concentration in the gel preparation reduced the number and length of the streaks, eventually disappearing at alginate concentrations above 5 wt.%. Figure 1b is a phase diagram showing patterns based on the preparation temperature and the alginate concentration used. The images in each region of the phase diagram display typical images taken at the respective phases. The concentric rings were formed under all conditions except for an alginate concentration of less than 1 wt% or above 80 °C. When the temperature was below 80 °C and the concentration was below 6 wt.%, stripe rings and radial streaks appeared simultaneously. We classified all samples into three types: 1. no pattern, 2. multiple-rings + radial streaks, and 3. concentric multiple-rings, as shown in Figure 1b. Figure 1c,d show the thumbnails of the contrast-enhanced images taken on (c) a black background to make the concentric stripe rings clearer and (d) a white background to highlight the radial streaks. The gels became more opaque at higher preparation temperatures and alginate concentrations. The light and shade contrast of the rings almost matched the thickness. The difference was enhanced in areas with high alginate concentrations and low temperatures. Thus, the formed patterns stayed almost permanently, even in a thermodynamically metastable state.

## 3. Discussion

**Figure 2:**
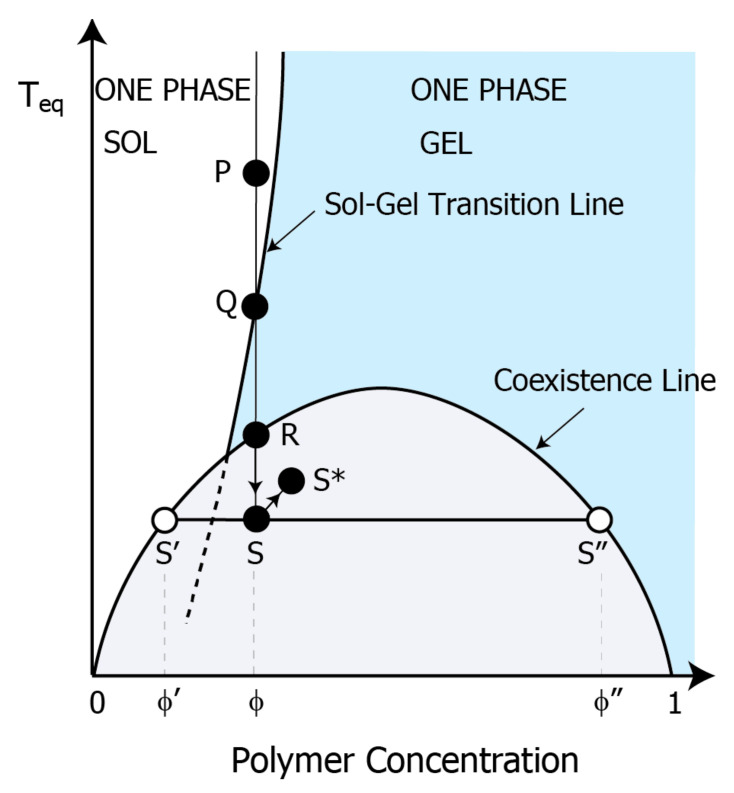
Phase diagram of the binary system undergoing gelation. Typical polysaccharide aqueous solution system with sol-gel and coexistence curve. The vertical and horizontal axes represent reduced temperature and polymer concentration, respectively. The upper area of the coexistence line represents one phase (polymer and solvent are in a state of dissolution). The area under the line is two phases (polymer-rich phase and poor phase coexist). φ indicates the prepared polymer concentration. φ′ and φ″ indicate the polymer concentration of the poor- and rich-phase, respectively. The blue-colored region below the sol-gel transition line is in a gel state. The increase in calcium ion concentration in this system is equivalent to a decrease in the reduced temperature.

The turbidity observed in the gel results from scattered light due to the microphase separation; the separated phases have individual refractive indices originating from each polymer concentration. The micro-separated domains are pinned, and they maintained their size before the separated domain grows macroscopically due to increased viscosity caused by alginate gelation [18]. Thus, formed patterns stay almost permanently, even in a thermodynamically metastable state. Figure 2 shows the equivalent phase diagram of a typical gel-forming system in a good solvent [16,26]. The vertical and horizontal axes represent the equivalent temperature and polymer concentration, respectively. This phase diagram shows that the gelation line lies above the binodal line. The addition of salts shifts the gelation and binodal lines upward due to a decrease in solubility resulting from neutralizing the charged alginate and complex formation. These upper shifts of gelation and phase separation allow for being cloudy near room temperature. In other words, adding salt to an ionic polysaccharide solution is nearly equivalent to temperature quenching. This phase diagram shows that dilute calcium nitrate solutions allow the alginate sol to gel without phase separation, i.e., P shifts to a position between Q and R. Thus, a transparent gel appears at room temperature over a wide range of alginate concentrations. As the salt concentration increases, the sol-gel transition line shifts to a position between Q and R; thus, the transparent gel becomes a white turbiditic gel due to microphase separation in the gel. Increasing the initial concentration of alginate, i.e., transferring S to S*, results in a number of polymer-rich domains, resulting in increased light scattering volume. This increase in polymer-rich domains is a reason for the turbidity enhancement at the higher alginate concentration, as shown in Figure 1c. At lower equivalent temperatures, the alginate solution is deeply quenched, making it unstable, and it is clearly separated into polymer-rich and poor phases, enhancing the refractive index difference. This high contrast causes strong turbidity at the lower temperatures in Figure 1c. Because this phase diagram can explain our resulting gel properties, we will discuss the stripe pattern based on gelation with phase separation. Based on the concept of phase separation and gelation, the formation of the concentric rings can be explained in the following scenario. The reaction of alginate and calcium ions forms the alginate–calcium complex. This complex is less soluble and condenses in water, causing nucleation when the complex concentration reaches its saturation concentration in water. According to the nucleation and growth theory, the nucleated alginate–calcium domain grows by collecting and aggregating the surrounding alginate–calcium polymer networks. In contrast, the alginate–calcium concentration in the surrounding area should decrease due to the alginate–calcium consumption caused by the growth of the first opaque ring via Ostwald ripening. This enrichment and dilution of the alginate polymer near the calcium diffusion edge has been observed in the formation of alginate gel particles formed by calcium ions [27,28]. This generation of alginate concentration gradient has also been demonstrated by some diffusion models for the gel front in the alginate gelation system [29,30]. Consequently, the outer ring of the first opaque ring becomes transparent and concave. Simultaneous gelation in this phase separation system suppresses the growth of phase separation and fixes the first opaque ring and its outer transparent ring; such transparent–opaque anisotropic structures triggered by phase separation and fixed by gelation have been similarly reported in gels formed from polysaccharide solutions by diffusion ions [1,12]. The reduced concentration of complexes in the outer region of the first opaque layer suppresses nucleation because its concentration is lower than the saturation concentration. Calcium ions diffuse through the medium successively, even during the gelation process, which forms similar cloudy and transparent ring pairs, mentioned above, outside of the transparent ring. By repeating this scenario, a concentric stripe pattern is formed. Such a banding structure resulting from a two-dimensional phase-separated system in two dimensions has been previously predicted by the phase separation of binary fluid confined within a narrow strip. We also note the convex and concave shape on the resultant alginate gel surface may be formed by the shrinking of alginate gel [31] or interaction with the mineral oil overlying the alginate layer [32,33]; the macroscopic heterogeneous structure induced by phase separation could ripple the aqueous solution-oil phase interface because of the different surface tension.

This system’s stripe formation is similar to a pattern known as the Liesegang (LG) patterning phenomenon [21]. The LG phenomenon is the spatial patterning process left by the final deposit C when electrolyte A (diffusant) diffuses into a gel containing another electrolyte B (reactant) to form product C. However, this system differs from the well-known Liesegang phenomenon in the number of performers to form; the LG phenomenon typically requires at least three components, including diffusant A, reactant B, and ready-made hydrogel. In the case of our patterning, the hydrogel is formed simultaneously through the patterning process, using only two performers.

**Figure 3:**
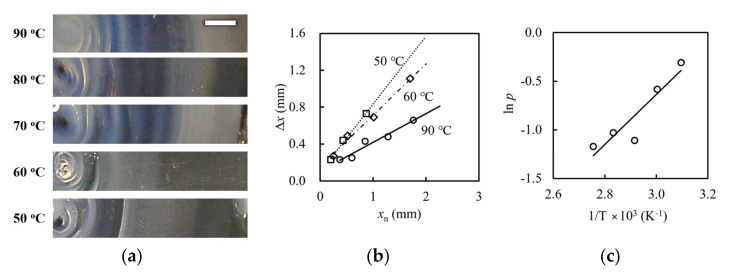
Temperature dependence of the calcium alginate gel pattern at 5 wt% alginate concentration: (**a**) magnified images of concentric stripes; (**b**) the relationship between the spacing *x*_n_ and its distance from the diffusing end *x*_n_ prepared at a given temperature; and (**c**) The relationship between the slope *p* and 1/*T*. The lines are the least-squares fit lines. The white bar represents 10 mm.

In the following, we will address the numerical similarity of our patterns to those of the Liesegang bands. The Liesegang band has some spatial regularity, and this rule is known as “spacing law” [22,23,24,25]. The law of spacing is that the ratio of the distance between adjacent bands (the location of the final deposit C) to the distance from that band to the diffusion edge is constant. This law is expressed as:

∆*x*_n_ = *p x*_n_ + const., (1)

where ∆*x*_n_ is the distance between two adjacent layers, and *x*_n_ represents the distance between the position of n th layer and the diffusing end. To compare numerically with the Liesegang phenomena, ∆*x*_n_ dependence for the *x*_n_ is focused on in Figure 3. Figure 3 also shows the temperature dependence of the alginate patterns at 5 wt% alginate. The magnified images cropped on one side from the center of the concentric bands obtained at different temperatures are shown in Figure 3a. The spaces between adjacent cloudy bands appear to narrow as the temperature increases. Figure 3b is the adjacent distance of bands of nth ∆*x*_n_ against the distance between the nth band and the calcium’s diffusion center *x*_n_. The relationship between the ∆*x*_n_ and *x*_n_ is roughly linear within the studied temperature range. These findings suggest that the spatial regulation in present experiments agrees with that of the Liesegang phenomena. Liesegang banding has been widely studied using nucleation in the presence of a moving front as the underlying mechanism [22,25]. The Liesegang phenomenon employed in the general theory consists of a chemical species B in a uniformly filled hydrogel of concentration *b*_0_ and another chemical species A of concentration *a*_0_ diffusing from the diffusion point. A chemically reacts with B and forms C. C increases with time, and, if the concentration reaches the saturated concentration, it becomes immobile precipitate D; A + B → C → D. Precipitate D appears after supersaturation. This nucleation threshold for precipitate growth kinetics has been proposed in several theories, depending on how it will be treated. According to a model interpreting the Liesegang phenomenon based on nucleation and growth theory, the coefficient of *p* in the spacing law can be described by the following equation [22]:

(2) $$p=F\left(b_{0}\right)+G\left(b_{0}\right)\frac{b_{0}}{a_{0}}\;\propto \;\frac{D_{a}}{D_{c}}\;,$$

where $D_{a}$ and $D_{c}$ are diffusion coefficients of A and B, respectively. *F* and *G* are decreasing functions of their argument $b_{0}$. This relationship demonstrates that *p* depends on the rate of diffusion coefficients $D_{a}/D_{c}$ in proportion. The diffusion constant can be expressed as D = *kT*/4*ηR*, where *k*, *T*, *η*, and *R* are the Boltzmann constant, the absolute temperature, the solution viscosity, and the hydrodynamic radius of the macromolecule, respectively. Thus, the ratio of diffusion coefficients be described as:

(3) $$D_{a}/D_{c}=\eta_{c}R_{c}/\eta_{a}R_{a},$$

The temperature dependence of viscosity is written by [34,35,36] *η* = exp(*E*/*kT*), where *E* is the activation energy; we confirm that the temperature dependence of the loss modulus G″ (Figure S2) demonstrated that the sodium alginate solution used in this study exhibits this property. Thus, the temperature dependences of *p* can be rewritten as:

*p* α exp (*E_c_* − *E_a_/kT*) (*R_c_/R_a_*) (4)

Equation (4) indicates that *p* is an exponential function of the inverse of the absolute temperature in proportion. To determine the dependence of *p* on temperature, we calculated the least-squares slopes of the lines from Figure 3b and obtained *p* at each temperature. Figure 3c shows ln *p* versus the inverse of temperature. We can see that ln *p* increases linearly with 1/*T*, suggesting that Equation (3) reasonably agrees with our system. The range of *p* calculated as 0.05–0.4 is also consistent with typical Liesegang phenomena. Therefore, the concentric ring patterns observed in the alginate–calcium nitrate system should be classified as a Liesegang phenomenon. Characteristic features of the Liesegang band have been found in spatial patterns of κ-carrageenan gel formed in a capillary tube by potassium chloride diffusing from the end of the capillary tube [26]. Because the elementary components and the system of κ-carrageenan-potassium chloride are basically the same as those presented here, Liesegang-like spatial patterns would form in diffusion-reaction systems that induce gelation of polysaccharide solution by diffusion of specific metal ions.

**Figure 4:**
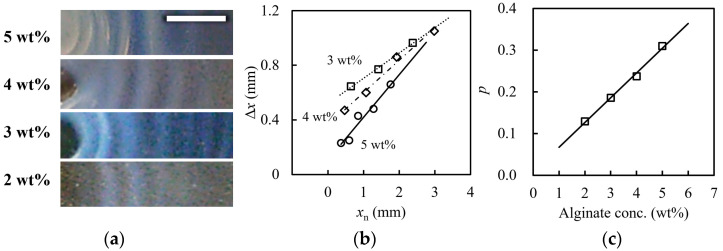
Alginate concentration dependence of the calcium alginate gel pattern at 90 °C in the preparation temperature: (**a**) magnified images of concentric stripes; (**b**) the relationship between ∆*x*_n_ and *x*_n_ prepared at given alginate concentrations; and (**c**) the relationship between *p* and the alginate concntration. The lines are the least-squares fit lines. The white bar represents 10 mm.

Figure 4 shows the alginate concentration dependence of the concentric stripe prepared at 90 ℃. From the magnified images of Figure 4a, we can see that these stripe intervals become wider with the distance from the center, and the white bands become clearer and thicker with increasing polymer concentration. The visual contrast of the pattern should be enhanced by the volume ratio of the separated phases and the phase concentration contrast, due to the refractive index difference between the separated phases. According to the phase separation scenario of Figure 2, high polymer concentrations allow a high-volume ratio of the polymer-rich phase to the poor phase: (φ″ – φ)/(φ – φ′). A high ratio enhances the shading contrast between the white and transparent bands. Figure 4b, which shows the relationship between *x*_n_ and ∆*x*_n_, indicates that the slope *p* increases approximately with alginate concentration; the values of *p* were 0.13, 0.19, 0.24, and 0.31 for alginate concentrations of 2, 3, 4, and 5 wt.%, respectively. Figure 4c illustrates the relationship between *p* and the alginate concentration. The *p* was linearly related to the alginate concentration in this range. According to Equation (2), the *p* is a linear function of the initial reactant concentration *b*_0_ in the typical Liesegang system. Thus, this alginate concentration dependence confirms that our concentric circle pattern is similar to the Liesegang pattern. High alginate concentrations rapidly consume the calcium ions diffusing from the center and then limit and reduce the diffusing calcium ions by forming the alginate–calcium complex. Thus, the limited calcium diffusion leads to a shallow quenching of the phase separation; the point S in the diagram shifts to S*, as shown in Figure 4. According to the Liesegang phenomenon based on phase separation theory, the space Δ*x* becomes narrower in deeper quenches where unstable phase separation occurs [22,25]. This quench depth becomes shallower with distance from the diffusion end, which explains the widening of the ∆*x*_n_ spacing with the distance *x*_n_. Higher alginate concentrations trap more calcium ions, allowing the number of calcium ions available for diffusion to decrease rapidly with the distance *x*_n_, thus resulting in the shallower quench illustrated in the Figure 4 phase diagram. Differences in the size of the bumps appearing in the pattern may be due to changes in the viscosity of the pre-gel solution, which is affected by temperature and concentration. This is because preparation temperature and concentration affect the sphericity and total volume loss of calcium alginate gel particles [20].

**Figure 5:**
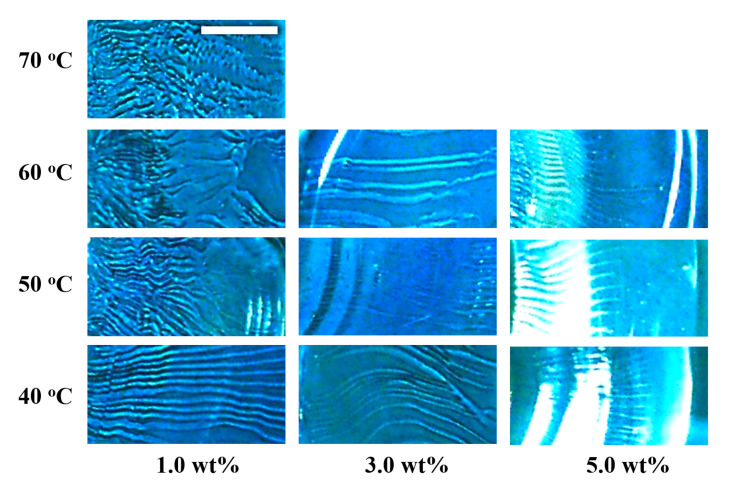
Magnified images of the radial pattern prepared at different temperatures and alginate concentrations. The white bar represents 10 mm.

Figure 5 shows the enlarged images of the radial patterns prepared at different temperatures and alginate concentrations. The radial lines are approximately parallel but not necessarily straight. Some lines have unique curves along with the inflated surface originating from the concentric rings. The radial patterns linearized and shorted at lower temperatures and higher alginate concentrations. The streak pattern does not appear in the alginate solution above 5 wt.%. The streaks that form the pattern are cracks on the hydrogel surface. The cracks arise and develop after forming the concentric ring pattern mentioned above; hence, the streaks were created after forming a weak hydrogel. For the pattern formation (for deformation) after gelation, more stress is required compared to that on the solution. Increasing the mechanical strength and the elastic modulus of the gel causes the gel to be more difficult to deform, and it also does not cleave. The storage modulus increases with concentration even in a sodium alginate solution (see Figure S2).

High elastic moduli at high alginate concentrations would explain why the streak pattern disappears at alginate concentrations above 6 wt.%; the high elasticity at high alginate concentrations discourages the formation of cracks. The storage modulus of sodium alginate solution also increases modestly with decreasing temperature, as shown in Figure S3. The increase in the elastic modulus with decreasing temperature would explain the disappearing streaks of 5 wt% alginate solution at 30 °C, although it forms at 50 °C. The non-formation of streaks at temperatures higher than 80 °C cannot be explained yet. The reason for the streak formation would be the gel shrinkage resulting from insolubilization due to the charge cancellation of alginate chains by reaction with calcium ions. The shrinkage after gelation would provide a uniform lateral tension on the circumference of the concentric circles. This lateral shrinkage tension on the circumferential will create cracks on the surface of the gel because surfaces have more mobility than the balk gel. The shrinkage stress becomes larger as the alginate polymer and water affinity decrease. Thus, high shrinkage stresses should be present in areas closer to the diffusion point where calcium ions are more concentrated, resulting in gel cracks starting closer to the diffusion point. The generated cracks would radially extend toward the rim, where the calcium ion concentration is lower and the breaking tension is weaker, farther away from the diffusion point. The result would be crack lines radiating from the diffusion point to the rim [37]. The cracks appearing on the gel surface may have similar kinetics of patterning cracks caused by the drying processes of protein solution drops [38], blood drops [39], colloidal silica [40], polymer films [41], and starch slurry [42]. This drying shrinkage process causes patterned cracks driven by drying to arrange themselves, and they are called shrinkage crack patterns. The parallel and nearly equidistant cracks are observed in inhomogeneous shrinkage due to directional drying from the edge down [43,44]. In our system, such inhomogeneous process gelation is developed from the center of the petri dish. Therefore, the parallel crack lines in a radial direction are drawn on the gel surface. The winding and disorder lines observed in the high-temperature region would be due to the increasing fluctuation of the edge where gelation is induced.

## 4. Conclusions

In alginate plate gels, formed by calcium ions diffusing toward the periphery, two characteristic patterns take shape. These are the concentric and radial patterns. The concentric patterns have the same spatial rules as the Liesegang pattern and could be explained as a pattern driven by phase separation occurring simultaneously with gelation. The radial pattern consists of surface cracks in the hydrogel. The required situation and the tendency toward equal spacing are similar to the horizontal banding formed in the drying process to monitor crack propagation. These patterns should be expected due to the competition between gelation and phase separation in the aqueous solution of a polysaccharide–metal ion system, driven by the diffusion of metal ions into the polysaccharide solution.

## 5. Materials and Methods

### 5.1. Materials

The samples of sodium alginate as the pre-gel solution and calcium nitrate (reagent grade) used as the gel-promoting agent were obtained from Wako Pure Chemical Industries Ltd. (Osaka, Japan) and were used without further purification. The molecular weight of the sample alginate was determined to be 1.7 × 10^5^ by the gel permeation chromatography.

### 5.2. Preparation of Sodium Alginate Pre-Gel Solution and Calcium Nitrate Solution

Sodium alginate solutions: sodium alginate was added to a screw-cap vial containing pure water (Milli-Q system, Merck Millipore, Tokyo, Japan) and a stirring tip. This mixture was immersed in a temperature-controlled water bath at 80 °C and stirred slowly in the bath with a magnetic stirrer for 3 days. In this way, a 6 wt% alginate solution was first prepared. After diluting these 6 wt% sodium alginate solutions with pure water, they were mixed well under the same conditions as above to obtain 2, 3, 4, and 5 wt% aqueous sodium alginate solutions. Calcium nitrate solution: calcium nitrate was diluted with pure water to obtain 6.0 M calcium nitrate solution as a gel-promoting agent.

### 5.3. Preparation of Alginate Gels in a Two-Dimensional System

A volume of 5 mL of the given alginate solution was poured into a glass petri dish with radii of 45 mm. Then, 3 mL of mineral oil was slowly poured into the petri dish covered with the alginate solution to form a two-layer solution with the mineral oil at the top and the alginate solution at the bottom. The mineral oil was used to prevent the water in the alginate solution from evaporating and changing the concentration, and it was completely separated from the alginate solution. Next, to form a gel, 4 µL of calcium nitrate solution was slowly added to the mineral oil solution layer in the center of the petri dish using a micropipette. Due to the geometric conditions, the gel-promoting ion Ca^2+^ diffuses through the alginate solution layer from the center to the limb of the petri dish. These operations were performed in a temperature-controlled bath with fixed temperatures from 40 °C to 90 °C in 10 °C increments, and they were left at each temperature for one day.

### 5.4. Observation and Analysis

All the resultant images were captured by a digital camera (QV-2300UX, Casio, Chiyoda City, Tokyo). To clearly visualize the macro-surface structures, the hydrogel images were captured using white or black papers as the background. Light intensity profiles in the space were obtained, and band spacing was analyzed using image analysis software (Image-Pro Plus 7.0.0, Media Cybernetics, Inc. Rockville, MD, USA).

## Supplementary Materials

The following supporting information can be downloaded at: https://www.mdpi.com/article/10.3390/gels9060444/s1, Figure S1: Higher quality images in Figure 1c,d. Figure S2: Loss moduli G″ of sodium alginate aqueous solution at various alginate concentrations and temperatures. Figure S3: Storage moduli G′ of sodium alginate aqueous solution at various alginate concentrations and temperatures.
