# Supplementary material for: Macroscopic Pattern Formation of Alginate Gels in a Two-Dimensional System
Source: Gels. 2023 May 26;9(6):444. doi: 10.3390/gels9060444 (PMC10297938; doi:10.3390/gels9060444)
Supplement: Supplementary file 1 [file gels-09-00444-s001.zip › gels-2397104-supplementary.pdf]

Supporting Information

Macroscopic pattern formation of alginate gels in a two-dimensional system

Ryota Haraguchi, Yushi Oishi and Takayuki Narita*

[1] Department of Chemistry and Applied Chemistry, Saga University, 1 Honjo, Saga, 840–8502, Japan; 22804003@edu.cc.saga-u.ac.jp (R.H.); oishiy@cc.saga-u.ac.jp (T.N.)
* Correspondence: naritat@cc.saga-u.ac.jp; Tel.: +81-952-28-8805

Contents

Figure S1: Higher quality images in Figs. 1(c) and (d).
Figure S2: Loss moduli G" of sodium alginate aqueous solution at various alginate concentrations and temperatures.
Figure S3: Storage moduli G′ of sodium alginate aqueous solution at various alginate concentrations and temperatures.

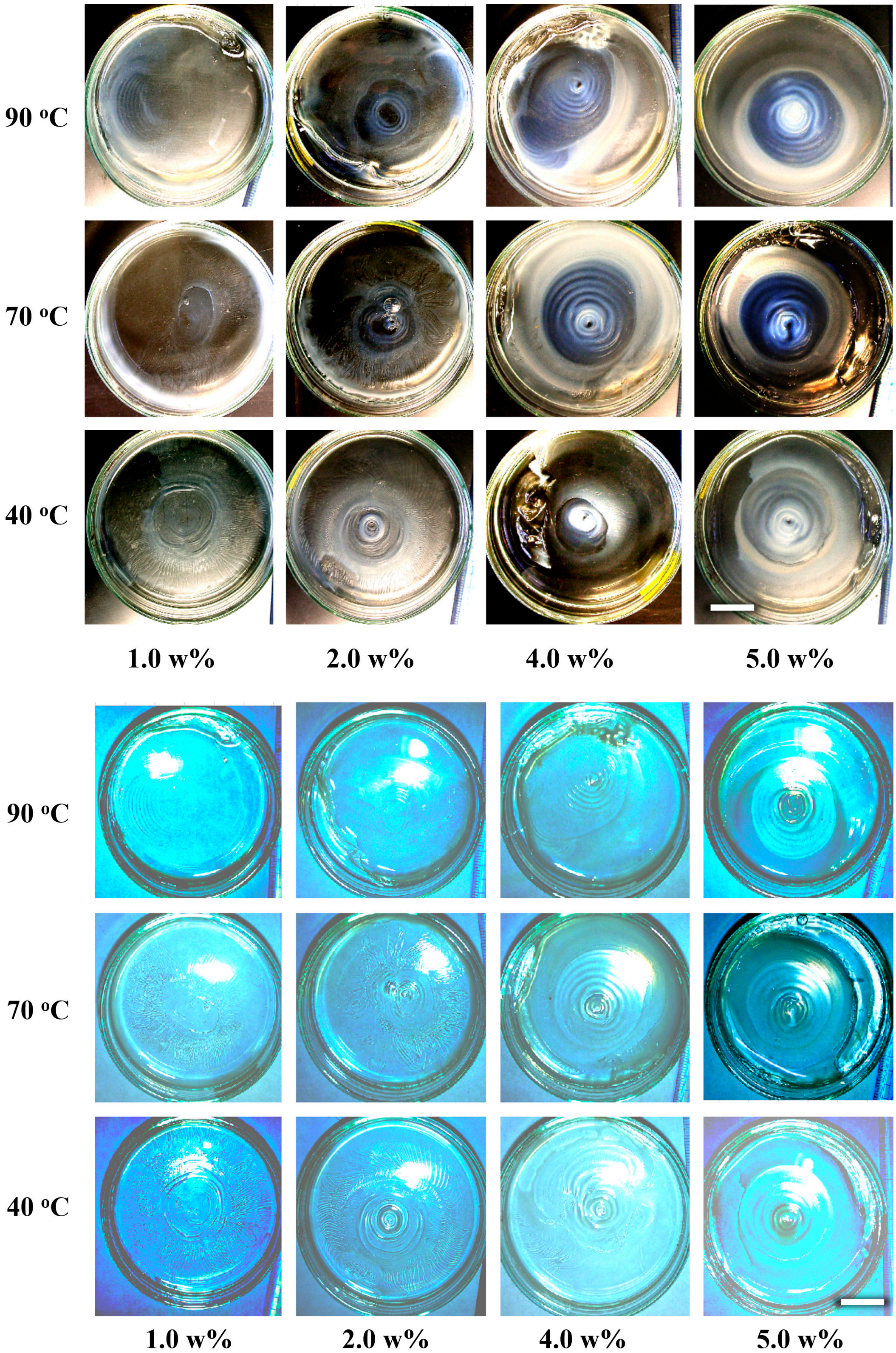

Figure S1: Higher quality images in Figs. 1(c) and (d).

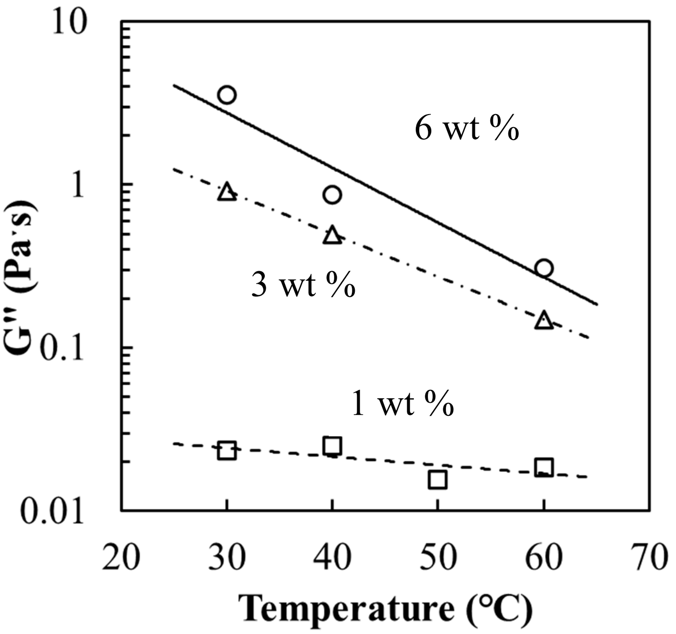

Figure S2. The effect of the alginate concentration and temperature on the loss modulus G" of sodium alginate solution. The G" values were determined using a rotary rheometer MCR-101 (Anton Paar, Graz, Austria) with plate-plate geometry (25 mm, 1 mm gap). Measurements were performed at given concentrations and temperatures controlled by pelche1 rad/s as the frequency with a strain value of 0.1%. The sample temperatures were controlled by a Peltier device at given temperatures.

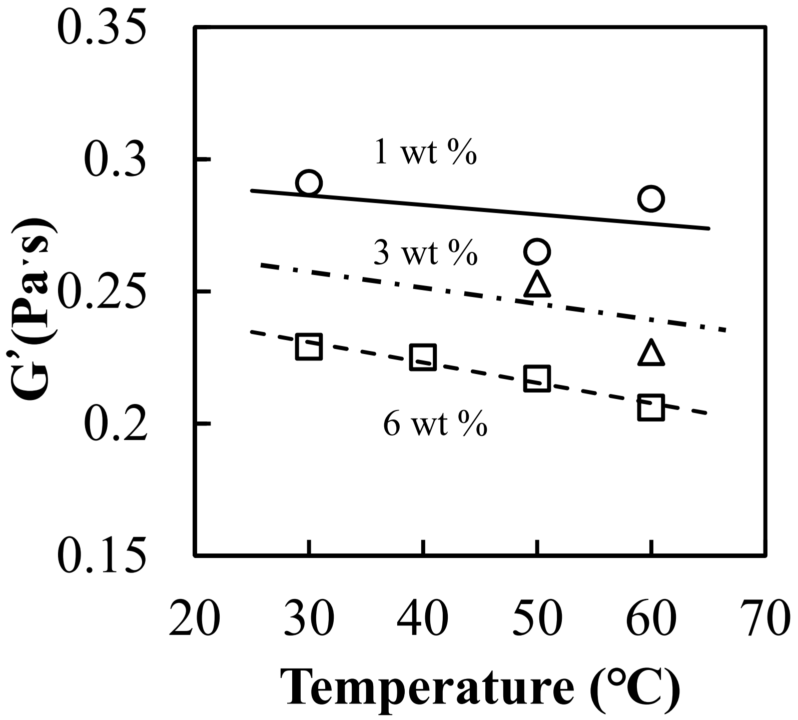

Figure S3. The effect of the alginate concentration and temperature on the storage modulus G′ in sodium alginate solution on the concentration and temperature. G′ were determined by the same method as Fig. S2.
